# Enhanced Circularly Polarized Green Luminescence Metrics from New Enantiopure Binary *Tris*-Pyrazolonate-Tb^3+^ Complexes

**DOI:** 10.3390/molecules29245887

**Published:** 2024-12-13

**Authors:** Jiaxiang Liu, Yongwen Zhang, Ruijuan Yao, Haitao Ren, Weijie Wang, Haohao Feng, Wentao Li, Zongcheng Miao

**Affiliations:** 1 Xi’an Key Laboratory of Advanced Photo-Electronics Materials and Energy Conversion Device, Technological Institute of Materials & Energy Science (TIMES), Xijing University, Xi’an 710123, China; 20230096@xijing.edu.cn (J.L.);; 2 School of Pharmacy, Jiangxi Science & Technology Normal University, Nanchang 330013, China; 3 Institute of Advanced Optoelectronic Materials and Technology, College of Big Data and Information Engineering, Guizhou University, Guiyang 550025, China; 4 School of Artificial Intelligence, Optics and Electronics (iOPEN), Northwestern Polytechnical University, Xi’an 710072, China

**Keywords:** chiral *tris*-pyrazolate-Tb^3+^ complexes, high-purity green luminescence, ligand-centered absorption behavior, circularly polarized luminescence brightness

## Abstract

Achieving superior circularly polarized luminescence brightness (*B*_CPL_) is an important subject and continuous challenge for chiroptical materials. Herein, by applying a binary molecular design for the synthesis of chiral organo-Tb^3+^ molecules, a novel pair of mononuclear chiral *tris*-pyrazolate-Tb^3+^ enantiomers, [Tb(PMIP)_3_(*R*,*R*-Ph-PyBox)] (**2**) and [Tb(PMIP)_3_(*S*,*S*-Ph-PyBox)] (**5**), have been synthesized and characterized. The three 1-phenyl-3-methyl-4-(isobutyryl)-5-pyrazolone (**HPMIP**) ligands play the role of efficient luminescence sensitizers and strong light-harvesting antennas, while the enantiopure 2,6-bis(4-phenyl-2-oxazolin-2-yl) pyridine ligand (***R*,*R***/***S*,*S*-Ph-PyBox**) is employed as the strong point-chiral inducer. With the proper combination of the **HPMIP** and **Chiral-Ph-PyBox** within the Tb^3+^ enantiomers, strong (**PMIP**)**^−^**-centered π-π^*^ electronic absorption (*ε*_263 nm_ = 38,400–39,500 M^−1^ cm^−1^) and brilliant high-purity ligand-sensitized Tb^3+^-centered green luminescence (*Φ*_PL_ = 47–48%) were observed. In addition, a clear circularly polarized luminescence (CPL) activity (|*g*_lum_| = 0.096–0.103) was also observed, resulting in a strong *B*_CPL_ (610–623 M^−1^ cm^−1^) for the two Tb^3+^ enantiomers from the hypersensitive transitions. Our results offer an effective path to develop high-performance chiroptical organo-Tb^3+^ luminophores.

## 1. Introduction

Circularly polarized luminescence (CPL) refers to a chiroptical phenomenon in which excited chiral luminophores emit left- or right-circularly polarized emissions after excitation with unpolarized light at a specific wavelength [1]. The chiroptical property is of particular interest owing to its fascinating potential applications in advanced anti-counterfeiting inks [2], enhanced information encryption [3], as well as high-resolution circularly polarized OLEDs (CP-OLEDs) [4]. The comprehensive CPL properties are normally characterized by the CPL brightness (*B*_CPL_; see Equation (1)) [5], and an ideal chiroptical luminophore should maximize four metrics as much as possible: the dissymmetry factor (*g*_lum_), overall luminescent quantum efficiency (*Φ*_PL_), molar extinction coefficients (*ε*_λ_) at the excitation wavelength, and branching ratio (*β*_i_; 0 ≤ *β*_i_ ≤ 1) of the considered emission peak. These metrics do not correlate, as they are governed by different selection rules [6]. Hence, it becomes a challenge to achieve a superior *B*_CPL_ value by maximizing all parameters for a specific CPL-active system.

(1) $$B_{CPL}={\;\beta }_{i}\times \varepsilon_{\lambda }\times \Phi_{PL}\times \frac{\left|g_{lum}\right|}{2}\;$$

An effective strategy to overcome this problem is to design chiral heteroleptic binary metal (transition/lanthanide) complexes [6,7,8], where two types of ligands with different functions can facilitate the synergistic optimization of each key metric simultaneously. The former often suffers from a relatively low degree of polarization (10^−3^ ≥ |*g*_lum_| ≥ 10^−5^) due to the inherent strong electric dipole (ED) transition moment with a weak magnetic dipole (MD) transition moment [9]. In contrast, significant CPL activity (|*g*_lum_| ≥ 10^−2^) can be observed in the latter with an allowed MD but forbidden ED character from the [Xe]4f^6^/4f^8^ electronic structure of most trivalent lanthanide ions (Ln^3+^ = Eu^3+^, Tb^3+^, Sm^3+^ and Dy^3+^, etc.) [10]. Meanwhile, the Ln^3+^-characteristic radiative transitions and light absorption capacity can be improved by the involvement of both singlet and triplet excitations simultaneously with a controllable antenna effect through the sensitizing ligands [11,12], achieving high *Φ*_PL_ and *ε*_λ_ values. Eu^3+^/Tb^3+^-based systems are regarded as some of the best candidates for achieving a large *B*_CPL_ value for circularly polarized monochromatic red/green luminescence due to their high intrinsic quantum efficiencies (*Φ*_Ln_) [13]. A number of successful examples of CPL-active heteroleptic binary Eu^3+^-centered red-light complexes have been reported in recent years, in which relatively high *B*_CPL_ values (103.7–161.73 M^−1^ cm^−1^) at ^5^D_0_→^7^F_1_ transitions were obtained through the coupling of point-chiral N^N/N^N^N-donor ligands to the well-developed chromophoric [Eu(*β*-diketonate)_3_(H_2_O)_x_] systems [14,15,16,17]. However, despite the concerted efforts in the synthesis of chiral Tb^3+^ complexes using a similar binary modular strategy, the relevant literature on heteroleptic binary Tb^3+^-based systems is very scarce and mainly limited to a few *tris*-*β*-diketonate-based and coumarin-based Tb^3+^ complexes [18,19,20,21,22] with point-chiral/planar-chiral auxiliary ligands with low *B*_CPL_ values (*B*_CPL_ ≤ 11.7 M^−1^ cm^−1^ at ^5^D_4_→^7^F_5_; see Table S1).

**Scheme 1:**
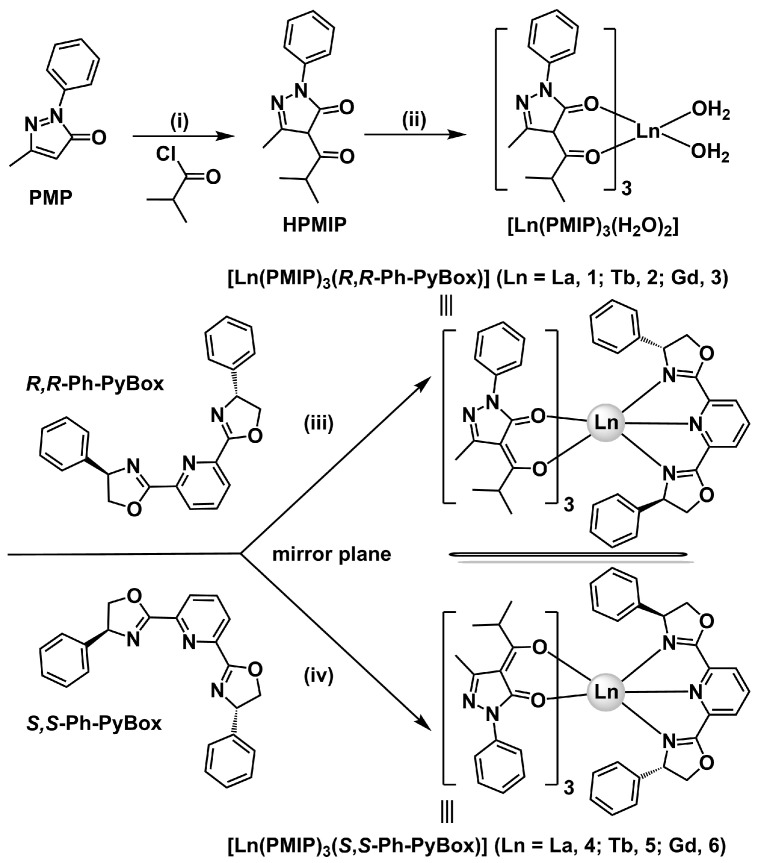
Synthetic route of the chiral organo-Ln^3+^ complexes **1**–**6**; experimental conditions: (i) 1,4-dioxane, Ca(OH)_2_, 70 °C, 2 h; (ii) CH_3_OH, NaOH, LnCl_3_·6H_2_O, 75 °C, 3 h; (iii) CH_3_OH, 95 °C, 12 h; (iv) CH_3_OH, 95 °C, 12 h.

Herein, we have combined a strong point-chiral auxiliary ligand, such as the commercially available enantiopure 2,6-bis(4-phenyl-2-oxazolin-2-yl) pyridine (***R*,*R*/*S*,*S*-Ph-PyBox**), with efficient sensitizing chromophores to increase the *B*_CPL_ of organo-Tb^3+^ molecules with a heteroleptic binary molecular design. We use pyrazolone 1-phenyl-3-methyl-4-(isobutyryl)-5-pyrazolone (**HPMIP**) as the chromophore, since it is one of the most widely used, highly efficient anionic organic antennae for sensitizing Tb^3+^-characteristic luminescence (generally, *Φ*_PL_ ≥ 30%) with strong π→π^*^ transitions located in the near-UV region [23,24,25]. The CPL properties of such a chiral pyrazolate-Tb^3+^ system have not been experimentally demonstrated yet. Here, we report the synthesis, characterization, photophysical properties, and theoretical calculations of a novel pair of mononuclear chiral nine-coordinate *tris*-pyrazolate-Tb^3+^ enantiomers, denoted as [Tb(PMIP)_3_(*R*,*R*-Ph-PyBox)] (**2**) and [Tb(PMIP)_3_(*S*,*S*-Ph-PyBox)] (**5**), respectively (see Scheme 1). In addition to the intense CPL activity (|*g*_lum_| = 0.096–0.103) associated with the excited-state chiroptical properties of the Tb^3+^ enantiomers, significantly enhanced *B*_CPL_ values (610–623 M^−1^ cm^−1^ at ^5^D_4_→^7^F_5_) of the high-purity green luminescence were also achieved. The analogous La^3+^/Gd^3+^ enantiomers were also synthesized to establish the liquid-state structure and the lowest electronic state energy levels of the chiral Ln^3+^ assemblies.

## 2. Results and Discussion

### 2.1. Synthesis and Characterization

Using the traditional self-assembly procedure in a solution, a series of precursor complexes [Ln(PMIP)_3_(H_2_O)_2_] (Ln = La, Tb, and Gd) were successfully prepared by the reaction of Lanthanide salts (LnCl_3_·6H_2_O) with **HPMIP** ligands in a 1:3 stoichiometry in the presence of NaOH. To introduce the optical and stereochemical chirality, the tridentate enantiopure bis(oxazoline) derivative ***R*,*R*-Ph-PyBox** or ***S*,*S*-Ph-PyBox** was introduced to fill the second coordination sphere of Ln^3+^ ions. The two series of compact saturated nine-coordinate chiral organo-Ln^3+^ complexes, [Ln(PMIP)_3_(*R*,*R*-Ph-PyBox)] (Ln = La, **1**; Tb, **2**; Gd, **3**) and [Ln(PMIP)_3_(*S*,*S*-Ph-PyBox)] (Ln = La, **4**; Tb, **5**; Gd, **6**), were obtained as colorless polycrystalline particles in high chemical yields (68–76%). The synthetic procedures of the pyrazolone ligand **HPMIP**, [Ln(PMIP)_3_(H_2_O)_2_] precursors, and corresponding chiral Ln^3+^ complexes **1**–**6** are given in Scheme 1.

**Figure 1:**
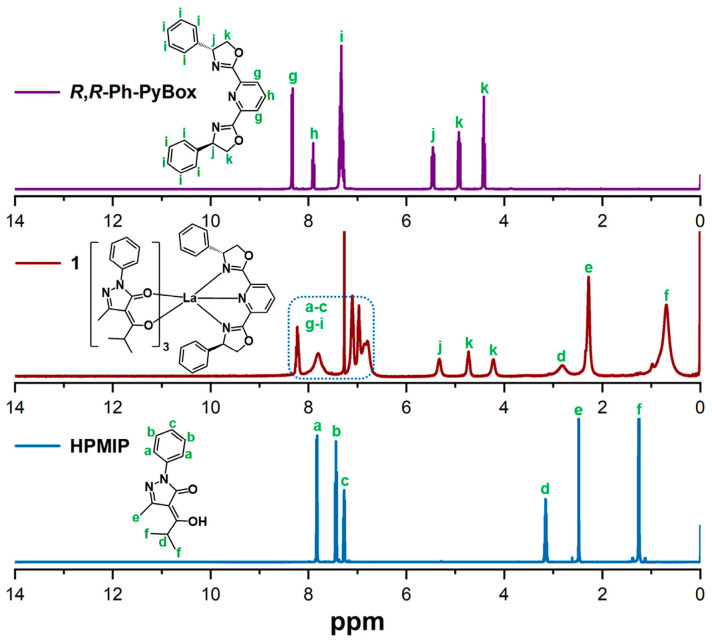
^1^H NMR spectra of the chiral La^3+^ complex **1** and associated free ***R*,*R*-Ph-PyBox** and **HPMIP** ligands in CDCl_3_ at room temperature.

The resulting **HPMIP** ligand, [Ln(PMIP)_3_(H_2_O)_2_] precursors, and chiral Ln^3+^ complexes **1**–**6** are fairly soluble in most common organic solvents (CH_3_OH, CH_3_CN, and CH_2_Cl_2_, etc.) and have been fully structurally characterized with a combination of EA, FTIR, ^1^H NMR, and MS spectroscopy techniques. The MALDI-TOF MS in positive-ion mode shows a series of parent peak signals with mass-to-charge ratios (*m*/*z*) of 1237.356, 1257.425, 1256.562, 1237.612, 1257.258, and 1256.651, which can unambiguously be assigned to single-charged cations [Ln(PMIP)_3_(Chiral-Ph-PyBox)]^+^ of **1**–**6**, respectively, indicating that the series of chiral Ln^3+^ complex molecules is structurally stable in a CH_3_CN solution. The ^1^H NMR spectra (400 MHz, CDCl_3_, 298 K) of the diamagnetic La^3+^ enantiomers and the associated free ligands are shown in Figure 1 and Figure S1. Relative to the free **HPMIP** (*δ* = 7.82–1.25 ppm) and **Chiral-Ph-PyBox** (*δ* = 8.34–4.41 ppm) ligands, the enantiopure La^3+^ assemblies exhibit a total of 12 significantly broadened resonances with moderate high-field shifts in the chemical shift range of 8.22–0.69 ppm. Additionally, the 3:1 integration ratio between the deprotonated **(PMIP)^−^** anionic ligands and the chiral auxiliary ***R*,*R*-Ph-PyBox** or ***S*,*S*-Ph-PyBox** ligand is consistent with the stoichiometry of the binary *tris*-pyrazolate-Tb^3+^ compound, [Ln(PMIP)_3_(Chiral-Ph-PyBox)], confirming the successful synthesis of the targeted Ln^3+^ molecule. Thermal analyses indicated that the two Tb^3+^ enantiomers have high decomposition temperatures (Δ*T*_5%_ = 272–274 °C; Figure S2), showing excellent thermal stability.

### 2.2. Ground-State Optical Properties

**Figure 2:**
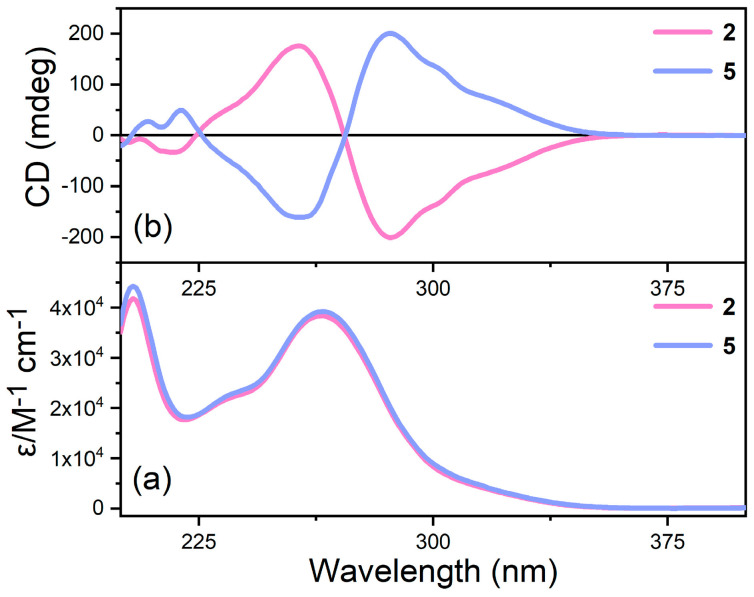
Ground-state optical property characterizations of **2** and **5**; (**a**) UV-vis absorption spectra; (**b**) CD spectra.

The UV−vis absorption and circular dichroism (CD) spectra of the free ligands and corresponding Tb^3+^/Gd^3+^ enantiomers were recorded in a chromatographic-grade CH_3_CN solution (*c* = 1.0 × 10^−5^ mol/L) at room temperature. Identical near-UV absorption behaviors (Figure 2a and Figure S3) for each pair of enantiomers (**Chiral-Ph-PyBox** and Tb^3+^/Gd^3+^ enantiomers) were observed, indicating that the chirality of the stereogenic centers that are localized at the bis(oxazoline) moieties has little influence on their ground-state photophysical properties [26]. Compared to the near-UV absorption bands (*λ*_abs_ = 234 and 266 nm; Figure S3) of free **HPMIP**, the two Tb^3+^ enantiomers also display a pair of ligand-centered absorption bands at 235 and 263 nm. The stronger lower-energy absorption band at 263 nm (*ε*_263 nm_ = 38,400–39,500 M^−1^ cm^−1^; see Table 1) is attributed to the singlet−singlet π-π^*^ enol absorption of the *β*-diketonate moieties in the deprotonated (**PMIP)^−^** ligands [27]. The blue shift (ca. 3 nm) of this band can be attributed to the decrease in the π-conjugation in the coupled **(PMIP)^−^** due to the twisted complexation of **(PMIP)^−^** to the Tb^3+^ ions [28]. As shown in Figure 2b, a pair of obvious mirror-imaged Cotton-effect bands ((+)-256 and (−)-286 nm for **2**; (−)-257 and (+)-285 nm for **5**) from the π-π^*^ electronic transitions of the achiral diketonate moieties of the **(PMIP)^−^** ligands were also observed, suggesting that the chirality was successfully introduced in the ground-state of the **(PMIP)^−^** ligands through the coordination of **Chiral-Ph-PyBox** to the achiral [Tb(PMIP)_3_(H_2_O)_2_] moiety [29].

### 2.3. Excited-State Optical Properties

**Figure 3:**
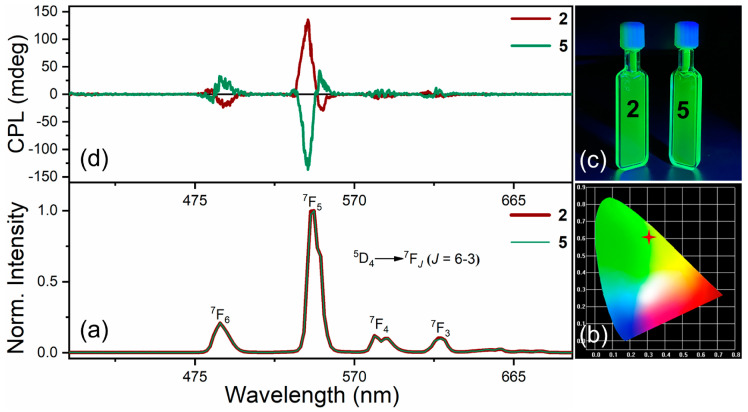
Excited-state optical property characterizations of **2** and **5**; (**a**) normalized PL spectra; (**b**) CIE 1931 chromaticity diagram; (**c**) photograph taken under 310 nm UV light; (**d**) CPL spectra.

The photoluminescence (PL) and CPL spectra of the Tb^3+^/Gd^3+^ enantiomers were measured under identical conditions as that for the ground-state absorption measurements. The excitation spectra of the two Tb^3+^ enantiomers show absorption peaks at ca. 330 nm (Figure S4), assigned to the excitation of the pyrazolone antennae moieties. The corresponding PL and CPL spectra in Figure 3 were recorded at ca. 330 nm excitation from enantiomer pairs **2** and **5**. The PL spectra (Figure 3a) clearly show a set of distinct Stark splitting narrow line-like bands at 490, 545, 585, and 621 nm, ascribed to the emissions from the ^5^D_4_ emissive state to the low-lying ^7^F*_J_* multiple states of Tb^3+^ ions with the corresponding *J* = 6–3, respectively. The absence of any residual ligand-based fluorescence emission in the expected energy range indicates a complete transfer of the absorbed energy from the excited π-states of the ligands (**(PMIP)^−^** and **Chiral-Ph-PyBox**) to the Tb^3+^ ions [30]. This energy transfer (ET) mechanism will be discussed later. The emission peaks associated with ^5^D_4_→^7^F_4,3_ transitions offer relatively low intensities, as they are partially or fully forbidden both in the MD or ED transitions [30]. In contrast, the stronger emissions based on the ^5^D_4_→^7^F_6_ and ^5^D_4_→^7^F_5_ transitions are ED- and MD-allowed, respectively. The observed hypersensitive ^5^D_4_→^7^F_5_ transitions (*β*_i_ = 67%; see Table 1) dominate the entire PL spectra from the two Tb^3+^ enantiomers, offering a brilliant Tb^3+^-centered high-purity green luminescence with Commission Internationale de l’Eclairage (CIE) chromaticity coordinates (Figure 3b,c) of *x* = 0.310–0.311 and *y* = 0.608. This bright PL luminous behavior is reflected by their corresponding high overall quantum yields (Figures S5 and S6) of 47% for **2** and 48% for **5**, which are almost comparable to the reported achiral *tris*-*β*-diketonate [31,32] and *tris*-pyrazolate Tb^3+^ complexes [23,24,25,33]. Significantly, they are much higher than any reported CPL-active heteroleptic Tb^3+^ emitter, with the exception of coumarin-based Tb^3+^ complexes (*Φ*_PL_ = 55%; Table S1) [22].

The time-resolved emission profiles of the hypersensitive ^5^D_4_→^7^F_5_ transition were characterized for the two Tb^3+^ enantiomers in a chromatographic-grade CH_3_CN solution (*c* = 1.0 × 10^−5^ mol/L) at room temperature. The decay curves were analyzed using a single exponential function, which gives the effective lifetime values of 798 and 795 μs for **2** and **5** (Figures S7 and S8). The excellent fitting with a single exponential decay function and μs lifetime indicates the involvement of one major monomeric configuration species in the solution with intrinsic long-lived phosphorescent properties [34,35]. The two Tb^3+^ enantiomers offer a pair of high-resolution quasi-mirror-imaged CPL profiles (Figure 3d) with positive–negative peaks related to the ^5^D_4_→^7^F*_J_* (*J* = 6–3) transitions, agreeing with the corresponding PL spectra. Both the electronic structure of the Ln^3+^ ion and the crystal field affect the CPL activity of the chiral Ln^3+^ compounds. The high *g*_lum_ values can usually be achieved for MD-allowed and ED-forbidden transitions [36]. The ^5^D_4_→^7^F_6,4,3_ transitions are expected to be less CPL-active, whereas the MD ^5^D_4_→^7^F_5_ transitions offer the most intense CPL bands, as confirmed from the CPL spectra in Figure 3d. This is also confirmed by the bisignate pattern of the *g*_lum_ values (Figure S9) of +0.103 and −0.096 for **2** and **5**, respectively. Such high *g*_lum_ values are comparable to the chiral PyBox-based Ln^3+^ complexes [14,17,19,37], representing one of the best chiroptical performance metrics from the organo-Tb^3+^ complexes using chiral–auxiliary ligands [18,19,20,21,38,39,40]. The relatively high *g*_lum_, combined with three other excellent optical metrics of *β*_i_, *ε*_λ_, and *Φ*_PL_, yield strong *B*_CPL_ values (Table 1) for the ^5^D_4_→^7^F_5_ transitions, reaching 623 and 610 M^−1^ cm^−1^ for **2** and **5**. To our knowledge, these *B*_CPL_ values represent the best performance for heteroleptic chiroptical Tb^3+^ emitters [13], as shown in Table S1.

**Figure 4:**
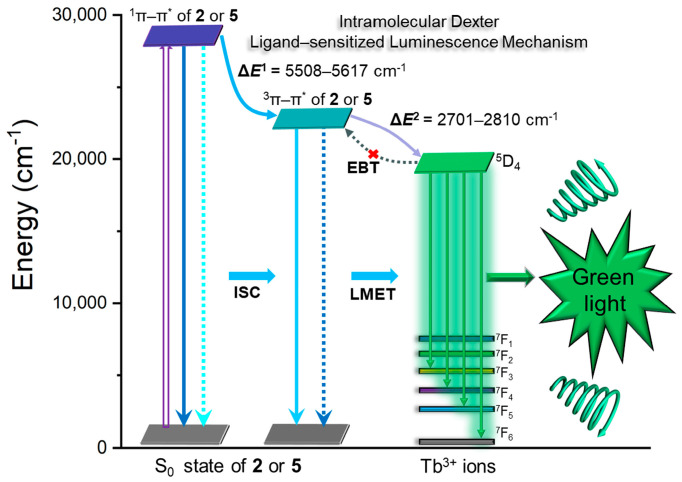
The relevant ^1^π-π^*^/^3^π-π^*^ energy levels and a possible intramolecular Dexter ET mechanism for **2** or **5**.

It is well established that the energy match within the organo-Tb^3+^ complexes is the key factor affecting luminescence behaviors. To elucidate the ET mechanism of the effective sensitization in our Tb^3+^ complexes, the energy levels of the relevant lowest excited states should be evaluated. Since the emission level of Gd^3+^ ion is very high (32,000 cm^−1^), the sensitization of the Gd^3+^ ion by an organic ligand is not possible. Therefore, the energy levels of the lowest triplet-excited state (^3^π-π^*^) of the luminescent Ln^3+^ complexes can be determined from the shorter-wavelength emission peaks (*λ*_em_ = 429 and 431 nm, respectively; Figure S10) from the corresponding isostructural Gd^3+^ complexes recorded at 77 K [41]. The ^3^π-π^*^ energy levels of complexes **3** and **6** are determined to be 23,310 and 23,201 cm^−1^, respectively, which are consistent with the theoretical calculation results (vide infra). The lowest singlet-excited state (^1^π-π^*^) energy levels of 28,818 cm^−1^ for **2** and **5** were estimated from their UV−vis absorbance edges (*λ*_edge_ = 347 nm) [42]. The relevant ^1^π-π^*^/^3^π-π^*^ energy levels, along with a possible intramolecular Dexter ET mechanism, are presented in Figure 4. In light of Reinhoudt’ and Latva’s empirical rules [43,44], an effective singlet–triplet intersystem crossing (ISC) process can be achieved due to the energy gaps between the ^1^π-π^*^ and ^3^π-π^*^, Δ*E*^1^ = *E*(^1^π-π^*^) − *E*(^3^π-π^*^) = 5508 and 5617 cm^−1^, for complexes **2** and **5**, which are slightly greater than 5000 cm^−1^. The energy differences between ^3^π-π^*^ and the first Tb^3+^ excited ^5^D_4_-emitting state at 20,500 cm^−1^, calculated from Δ*E*^2^ = *E*(^3^π-π^*^) − *E*(^5^D_4_), are 2701 and 2810 cm^−1^ for complexes **5** and **2,** indicating that the ET process from ligand to Tb^3+^ (LMET; ^3^π-π^*^→^5^D_4_) is possible, while there is no reverse energy transfer (EBT; ^5^D_4_→^3^π-π^*^) from the ^5^D_4_-emitting level to the ^3^π-π^*^.

### 2.4. Theoretical Calculations of Electronic Structure

**Figure 5:**
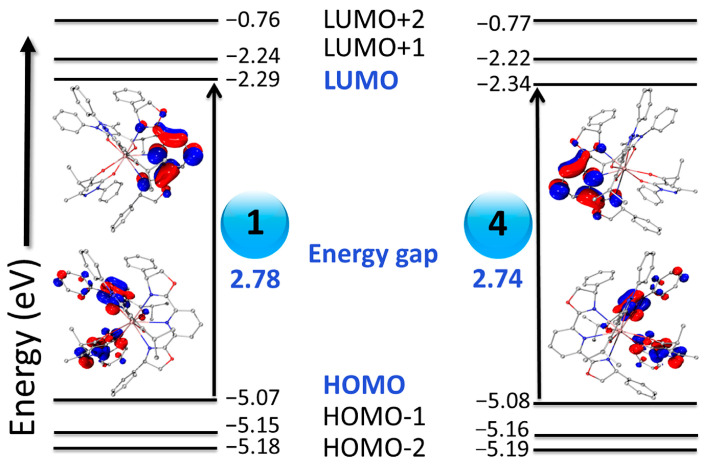
The frontier molecular orbitals’ energy levels and the HOMO and LUMO distributions for **1** and **4** are based on the optimized ground-state (S_0_) geometries.

To establish the photophysical properties of the two chiral Tb^3+^ enantiomers, density functional theory (DFT) calculations of the diamagnetic La^3+^ enantiomers were carried out based on the Becke–Lee–Yang–Parr (B3LYP) hybrid function because of the presence of uncoupled f electrons for Tb^3+^ ions [45,46]. The resultant minimum-energy structures, along with their respective frontier molecular orbitals HOMOs and LUMOs’ energy levels and electron density distributions, are shown in Figure 5 and Table S2. Moreover, time-dependent DFT (TD-DFT) calculations of the DFT-optimized structures were also performed to simulate the UV-vis absorption behavior of the Ln^3+^ enantiomers. The calculated absorption wavelengths (*λ*/nm), oscillator strengths (*f*), electronic vertical excitation energies (*E*/eV), transition types, and property details were established by interfragment charge transfer (IFCT) analysis and are summarized in Table S3.

It can be clearly seen that the orbital density distributions of the HOMOs and LUMOs in two chiral La^3+^ enantiomers are not affected by the participation of enantiopure ***R*,*R*-Ph-PyBox**, or ***S*,*S*-Ph-PyBox** ligands with nearly identical distribution patterns (see Figure 5 and Table S2). The LUMO distributions for **1** (98.09%) and **4** (98.05%) are almost exclusively localized on the **Chiral-Ph-PyBox** ligands. The corresponding HOMO distributions of complexes **1** and **4** are localized at the **(PMIP)^−^-1** ligand with ca. 44.03 and 45.02%, respectively. Meanwhile, the **(PMIP)^−^-2** constitutes 53.41 and 53.23% of complexes **1** and **4**, respectively. The theoretical HOMO and LUMO energy levels are in good agreement with the experimentally determined energy levels (*E*_HOMO_ = −5.44/−5.43 eV; *E*_LUMO_ = −1.87/−1.86 eV) that were estimated from the optical HOMO-LUMO gap values (*E*_g_^opt^ = 3.57 eV) and the oxidation potentials (*E*_ox_^onset^ = 1.15–1.16 V) from the cyclic voltammogram (versus an Fc/Fc^+^ couple; Figure S11 and Table S4). According to the TD-DFT calculations of the S_0_→S_1_ excitation (Table S3), the lowest-energy absorption bands in the UV-vis spectra of complexes **1**–**6** can be attributed to the singlet ligand-to-ligand charge transfer (^1^LLCT) with a dominant HOMO→LUMO (97.47% for **1** and 96.38% for **4**), mainly involving (**PMIP)^−^-1**→**Chiral-Ph-PyBox** and (**PMIP)^−^-2**→**Chiral-Ph-PyBox** mixed-transition contributions. In the calculated lowest-energy vertical S_0_→T_1_ transitions, two theoretical lowest triplet-excited state energy levels of 22,779 cm^−1^ for **1** and 22,831 cm^−1^ for **4** were calculated, which are close to the experimental values (23,201 and 23,310 cm^−1^) that were estimated from corresponding phosphorescence spectra of Gd^3+^ enantiomers, measured at 77 K.

## 3. Materials and Methods

### 3.1. General Information and Synthesis of Intermediates

Analytically pure and chromatographic-grade solvents were used for synthesis and spectral measurements, respectively. All solvents were purchased from Tianjin Fuyu Fine Chemical Co. (Tianjin, China). 1-phenyl-3-methyl-5-pyrazolone (**PMP**), isobutyryl chloride, ***R*,*R*-Ph-PyBox**, ***S*,*S*-Ph-PyBox,** and trivalent Lanthanide chloride hexahydrate salts (99.99% for LaCl_3_·6H_2_O, TbCl_3_·6H_2_O, and GdCl_3_·6H_2_O) were purchased from Aladdin (Shanghai, China). NaOH, Ca(OH)_2_, and concentrated hydrochloric acid (HCl, 37%) were supplied by Tianjin Damao Chemical Reagent Factory (Tianjin, China). The pyrazolone **HPMIP** ligand was synthesized by nucleophilic substitution of **PMP** with isobutyryl chloride in excess Ca(OH)_2_, according to our previous report [47,48]. The Ln^3+^ dihydrate complex precursors [Ln(PMIP)_3_(H_2_O)_2_] (Ln = La, Tb, and Gd) were synthesized using a traditional solution-phase self-assembly procedure [49,50]. Additional details about the synthetic procedures and the characterization of the intermediates (**HPMIP** and Ln^3+^ precursors) and spectroscopic measurement details are provided in the Supplementary Materials.

### 3.2. Synthetic Procedures and Characterizations of the Chiral Organo-Ln^3+^ Complexes ***1**–**6***

The equimolar amounts of the precursors [Ln(PMIP)_3_(H_2_O)_2_] (0.3 mmol; Ln = La, 0.271 g; Tb, 0.277 g; Gd, 0.277 g) and enantiopure tridentate N^N^N-donor ***R*,*R*-Ph-PyBox** (0.111 g, 0.3 mmol) or ***S*,*S*-Ph-PyBox** (0.111 g, 0.3 mmol) ligand were dissolved in CH_3_OH (30 mL) in a reaction flask. Each reaction mixture was stirred at 95 °C for 12 h. The resulting solution was left at an ambient temperature for crystallization, and the colorless polycrystalline products of **1**–**6** were obtained after the solution had evaporated for 3–4 days.

The analysis data for **1** are as follows: Yield = 0.260 g, 70%. Anal. Calcd. for C_65_H_64_N_9_O_8_La: C, 63.05; H, 5.21; N, 10.18%. Found: C, 63.11; H, 5.17; N, 10.26%. FTIR (KBr, cm^−1^): 3439 (w), 2961 (w), 2920 (w), 2859 (w), 1619 (vs), 1587 (m), 1533 (w), 1493 (s), 1443 (m), 1365 (m), 1317 (w), 1267 (w), 1236 (w), 1183 (w), 1138 (w), 1089 (w), 1064 (w), 1024 (w), 980 (m), 920 (m), 830 (w), 755 (m), 695 (w), 650 (m), 614 (w), 545 (w), 507 (w), 429 (w). ^1^H NMR (CDCl_3_, 400 MHz, 298 K): *δ* (ppm) 8.22 (m, 2H, -Py), 8.21 (t, 1H, -Py), 7.80 (s, 6H, -Ph), 7.09 (s, 7H, -Ph), 6.96 (t, 5H, -Ph), 6.82 (d, 7H, -Ph), 5.32 (d, 2H, -H^j^), 4.72 (t, 2H, -CH_2_ of -H^k^), 4.21 (s, 2H, -CH_2_ of -H^k^), 2.80 (s, 3H, -H^d^), 2.27 (s, 9H, -H^e^), 0.69 (s, 18H, -H^f^). MALDI-TOF MS: *m*/*z* 1237.356 (100%), [M-H]^+^.

The analysis data for **2** are as follows: Yield = 0.276 g, 73%. Anal. Calcd. for C_65_H_64_N_9_O_8_Tb: C, 62.05; H, 5.13; N, 10.02%. Found: C, 62.12; H, 5.17; N, 9.97%. FTIR (KBr, cm^−1^): 3446 (w), 2964 (w), 2921 (w), 2860 (w), 1619 (vs), 1586 (m), 1532 (w), 1491 (s), 1444 (m), 1364 (m), 1318 (w), 1267 (w), 1237 (w), 1183 (w), 1138 (w), 1089 (w), 1064 (w), 1022 (w), 979 (m), 919 (m), 830 (w), 754 (m), 695 (w), 651 (m), 614 (w), 546 (w), 506 (w), 428 (w). MALDI-TOF MS: *m*/*z* 1257.425 (100%), [M-H]^+^.

The analysis data for **3** are as follows: Yield = 0.278 g, 74%. Anal. Calcd. for C_65_H_64_N_9_O_8_Gd: C, 62.13; H, 5.13; N, 10.03%. Found: C, 62.18; H, 5.01; N, 10.08%. FTIR (KBr, cm^−1^): 3441 (w), 2960 (w), 2921 (w), 2858 (w), 1619 (vs), 1588 (m), 1534 (w), 1492 (s), 1443 (m), 1365 (m), 1318 (w), 1268 (w), 1235 (w), 1182 (w), 1139 (w), 1090 (w), 1063 (w), 1025 (w), 980 (m), 919 (m), 828 (w), 755 (m), 696 (w), 649 (m), 614 (w), 546 (w), 507 (w), 427 (w). MALDI-TOF MS: *m*/*z* 1256.562 (100%), [M-H]^+^.

The analysis data for **4** are as follows: Yield = 0.252 g, 68%. Anal. Calcd. for C_65_H_64_N_9_O_8_La: C, 63.05; H, 5.21; N, 10.18%. Found: C, 63.13; H, 5.27; N, 10.12%. FTIR (KBr, cm^−1^): 3442 (w), 2959 (w), 2920 (w), 2859 (w), 1618 (vs), 1586 (m), 1532 (w), 1494 (s), 1444 (m), 1367 (m), 1317 (w), 1267 (w), 1236 (w), 1182 (w), 1138 (w), 1089 (w), 1064 (w), 1023 (w), 979 (m), 918 (m), 830 (w), 755 (m), 695 (w), 649 (m), 615 (w), 546 (w), 506 (w), 428 (w). ^1^H NMR (CDCl_3_, 400 MHz, 298 K): *δ* (ppm) 8.22 (m, 2H, -Py), 8.20 (t, 1H, -Py), 7.80 (s, 6H, -Ph), 7.10 (s, 7H, -Ph), 6.96 (t, 5H, -Ph), 6.82 (d, 7H, -Ph), 5.32 (d, 2H, -H^j^), 4.72 (t, 2H, -CH_2_ of -H^k^), 4.21 (s, 2H, -CH_2_ of -H^k^), 2.80 (s, 3H, -H^d^), 2.28 (s, 9H, -H^e^), 0.69 (s, 18H, -H^f^). MALDI-TOF MS: *m*/*z* 1237.612 (100%), [M-H]^+^.

The analysis data for **5** are as follows: Yield = 0.286 g, 76%. Anal. Calcd. for C_65_H_64_N_9_O_8_Tb: C, 62.05; H, 5.13; N, 10.02%. Found: C, 62.14; H, 5.15; N, 9.95%. FTIR (KBr, cm^−1^): 3445 (w), 2963 (w), 2919 (w), 2860 (w), 1618 (vs), 1587 (m), 1533 (w), 1494 (s), 1443 (m), 1364 (m), 1317 (w), 1267 (w), 1237 (w), 1183 (w), 1138 (w), 1089 (w), 1064 (w), 1025 (w), 981 (m), 919 (m), 829 (w), 754 (m), 695 (w), 650 (m), 614 (w), 546 (w), 508 (w), 427 (w). MALDI-TOF MS: *m*/*z* 1257.258 (100%), [M-H]^+^.

The analysis data for **6** are as follows: Yield = 0.271 g, 72%. Anal. Calcd. for C_65_H_64_N_9_O_8_Gd: C, 62.13; H, 5.13; N, 10.03%. Found: C, 62.16; H, 5.02; N, 10.11%. FTIR (KBr, cm^−1^): 3440 (w), 2960 (w), 2919 (w), 2859 (w), 1620 (vs), 1586 (m), 1533 (w), 1492 (s), 1444 (m), 1364 (m), 1316 (w), 1267 (w), 1236 (w), 1183 (w), 1139 (w), 1089 (w), 1064 (w), 1024 (w), 981 (m), 920 (m), 831 (w), 756 (m), 694 (w), 649 (m), 614 (w), 544 (w), 507 (w), 429 (w). MALDI-TOF MS: *m*/*z* 1256.651 (100%), [M-H]^+^.

## 4. Conclusions

In conclusion, the coupling of an enantiopure bis(oxazoline) derivative (***R*,*R*-Ph-PyBox** or ***S*,*S*-Ph-PyBox**) successfully induced CD and CPL properties in well-developed chromophoric [Tb(PMIP)_3_(H_2_O)_2_] systems. This led to the design and synthesis of the first example of CPL-active pyrazolonate-Tb^3+^ molecules with bright Tb^3+^-centered high-purity green emission (*Φ*_PL_ = 47–48%) and intense excited-state chiroptical activity (|*g*_lum_| = 0.096–0.103). The diastereopurity of the complexes (**2** or **5**) in a solution was confirmed through various techniques, including the chiroptical spectra, single exponential decay responses, and hydrogen proton resonance distribution. Optical characterizations and theoretical calculations highlighted that coordinating two types of ligands with distinct functions to the Tb^3+^ ions enhances the antenna-centered light absorption in the π-π^*^ electronic transition range and conveys a one-way complete energy to the central Tb^3+^ ions. By balancing key metrics (*β*_i_, *ε*_λ_, *Φ*_PL_ and *g*_lum_), the highest *B*_CPL_ (up to 623 M^−1^ cm^−1^ for **2**) was achieved for the CPL-active heteroleptic Tb^3+^ emitters. This promising outcome suggests that further structural optimization of heteroleptic binary Ln^3+^ molecules could further enhance their CPL properties, delivering a higher *B*_CPL_ value for circularly polarized monochromatic green luminescence.

## Figures and Tables

**Table 1:** Summary of the circularly polarized green luminescence metrics for **2** and **5**.

Compound	*λ*_em_ (nm)	*ε*_263 nm_ (M^−1^ cm^−1^)	*Φ*_PL_	Transition	|*g*_lum_|	*β*_i_	*B*_CPL_ (M^−1^ cm^−1^) ^a^
**2**	490	38,400	47%	^5^D_4_→^7^F_6_	0.013	0.15	18
	545			^5^D_4_→^7^F_5_	0.103	0.67	623
**5**	490	39,500	48%	^5^D_4_→^7^F_6_	0.015	0.15	21
	545			^5^D_4_→^7^F_5_	0.096	0.67	610

^a^ Calculated using the following formula: *B*_CPL_ = *β*_i_ × *ε*_λ_ × *Φ*_PL_ × |*g*_lum_|/2 (details of the *β*_i_ and *ε*_λ_ metrics are given in the Supplementary Materials).

## Data Availability

All data are included in the article.

## Supplementary Materials

The following supporting information can be downloaded at https://www.mdpi.com/article/10.3390/molecules29245887/s1: Figure S1: ^1^H NMR spectra of complex **4** and associated free ***S*,*S*-Ph-PyBox** and **HPMIP** ligands in CDCl_3_; Figure S2: Thermogravimetric profiles of complexes **2** and **5**; Figure S3: UV-vis absorption spectra of the intermediates (**HPMIP** and **Chiral-Ph-PyBox**) and complexes **3** and **6,** recorded in CH_3_CN; Figure S4: Excitation spectra of complexes **2** and **5,** recorded by monitoring the emission bands of Tb^3+^ ions at 545 nm in CH_3_CN; Figures S5 and S6: Luminescent absolute quantum yield of complexes **2** and **5** based on the overall ^5^D_4_→^7^F*_J_* (*J* = 6–0) transition; Figures S7 and S8: The decay curves of complexes **2** and **5** in CH_3_CN, monitored at 545 nm; Figure S9: The *g*_lum_ profiles of complexes **2** and **5,** recorded in CH_3_CN; Figure S10: Excitation and emission spectra of complexes **3** and **6,** recorded in CH_3_CN; Figure S11: The CV curves of complexes **2** and **5,** recorded as a function of Fc/Fc^+^ in CH_3_CN; Table S1: A comparison of the circularly polarized green luminescence metrics recorded in this work with previously reported chiral heteroleptic binary Tb^3+^ emitters; Tables S2 and S3: Tabulated DFT and TD-DFT calculation results; Table S4: Summary of voltammetric data of studied chiral complexes **2** and **5**. References [13,18,19,20,21,22,47,51,52,53,54] are cited in the supplementary materials.
