# Supplementary material for: Enhanced Circularly Polarized Green Luminescence Metrics from New Enantiopure Binary Tris-Pyrazolonate-Tb3+ Complexes
Source: Molecules. 2024 Dec 13;29(24):5887. doi: 10.3390/molecules29245887 (PMC11676078; doi:10.3390/molecules29245887)
Supplement: Supplementary file 1 [file molecules-29-05887-s001.zip › molecules-3345802-supplementary.pdf]

Supporting Information

Enhanced Circularly Polarized Green Luminescence Metrics from New Enantiopure Binary *Tris*-Pyrazolonate-Tb^{3+} Complexes

Jiaxiang Liu [1], Yongwen Zhang [2], Ruijuan Yao [1], Haitao Ren [1], Weijie Wang [1], Haohao Feng [1], Wentao Li [3,*] and Zongcheng Miao [1,4,*]

[1]Xi'an Key Laboratory of Advanced Photo-Electronics Materials and Energy Conversion Device, Technological Institute of Materials & Energy Science (TIMES), Xijing University, Xi'an 710123, China; 20230096@xijing.edu.cn (J.L.)

[2]School of Pharmacy, Jiangxi Science & Technology Normal University, Nanchang 330013, China

[3]Institute of Advanced Optoelectronic Materials and Technology, College of Big Data and Information Engineering, Guizhou University, Guiyang 550025, China

[4]School of Artificial Intelligence, Optics and Electronics (iOPEN), Northwestern Polytechnical University, Xi'an 710072, China

*Correspondence: wtli@gzu.edu.cn (W.L.); miaozongcheng@nwpu.edu.cn (Z.M.)

Spectroscopic Measurements.

Structural Measurements. Elemental analyses (C, H, and N) were carried out on an elemental analyzer (Model: VARIO EL, Elementar, Hanau, Germany). Fourier transform infrared (FTIR) spectra were recorded on IR-4000 (JASCO International Co., Ltd., Tokyo, Japan) in the region of 4000 to 400 cm^{-1}. The hydrogen nuclear magnetic resonance spectra (^1H NMR) were recorded on a JEOL JNM-ECZ400S nuclear magnetic resonance spectrometer (Japan Electronics Corporation, 400 MHz) with tetramethylsilane (TMS) as the internal standard in $CDCl_3$ at 298 K, and the spectra assignments are reported as follows: s = singlet, d = doublet, t = triplet, m = multiplet. The mass spectra were recorded on a 4800 Plus MALDI TOF/TOF Analyzer (Applied Biosystems).

Optical and Chiroptical Measurements. UV-vis absorption spectra were recorded on a PerkinElmer LAMBDA 950 UV-Vis spectrophotometer (PerkinElmer, Waltham, MA, USA). The optical HOMO-LUMO gap values (E_g^{opt}) and lowest single-excited state ($^1\pi$-π^*) energy levels of the two Tb^{3+} enantiomers were estimated by referring to their respective wavelengths of UV−vis absorbance edges (λ_{edge}). The molar extinction coefficients of the lowest-energy absorption bands were calculated using the Beer-Lambert equation ($A = \varepsilon \times c \times l$; where A represents sample absorbance, c denotes sample concentration and l is the cuvette inner diameter) [1]. Excitation and emission spectra were recorded using an FLS 1000 spectrofluorometer (Edinburgh Instruments, UK). The integral proportion of emission intensity for each specific transition (the so-called branching ratio β_i; $0 \le \beta_i \le 1$) was calculated using a general

equation ($\beta_i = I_i/(\Sigma_j\ I_j)$) [2], where I_i represents the integrated intensity of the considered transition and $\Sigma_j\ I_j$ is the summation of the integrated intensities over all the transitions. Luminescence lifetimes were recorded on the FLS 980 (Edinburgh Instruments, UK) transient fluorescence spectrometer, and the decay curves for the two chiral Tb^{3+} enantiomers were well-fitted to be the single exponential function to provide the characteristic luminescence lifetimes. Photoluminescence quantum yields (Φ_{PL}) were determined using an FLS 1000 absolute photoluminescence quantum yield measurement system with an integrating sphere as the sample chamber. Circular dichroism (CD) spectra were measured on a JASCO J-810 circular dichroism spectrometer (JASCO International CO., Tokyo, Japan). The CPL spectra were measured on a Jasco CPL-300 spectrophotometer using 'Continuous' scanning mode at 100 nm/min scan speed. The test adopted "band" mode with an Ex and Em slit width of 16 nm and a digital integration time (D.I.T.) of 4.0 s with multiple accumulations (4 times); the g_{lum} values were read from the curves recorded using the equipment software bundle. The color chromaticity coordinates of the two chiral Tb^{3+} enantiomers were calculated using the CIE system.

Thermal Measurements. Thermogravimetric (TG) analysis was performed using a Q500 thermogravimetric analyzer (TA instruments, USA) under a nitrogen flow of 25 mL/min at a heating rate of 10 °C/min from 25-800 °C.

Electrochemical Measurements. Cyclic voltammetry (CV) measurements were conducted on an Autolab PGSTAT302N unit using degassed CH_3CN solution at room temperature in a N_2 atmosphere (scan rate = 100 mV/s). A conventional

three-electrode configuration consisting of a glassy carbon working electrode, a Pt-wire counter electrode, and a Ag/AgCl reference electrode was used. The supporting electrolyte was 0.1 M $[Bu_4N]PF_6$ in CH_3CN. Ferrocene was added as a calibrant, and all potentials reported are quoted with reference to the ferrocene-ferrocenium (Fc/Fc^+) couple.

DFT and TD-DFT Theoretical Calculations. In order to further elucidate the photophysical properties of the two chiral Tb^{3+} enantiomers and to support their electrochemical properties, density functional theory (DFT) calculations of diamagnetic La^{3+} enantiomers were carried out employing three-parameter Becke–Lee–Yang–Parr (B3LYP) hybrid function theory in Gaussian 09 [3]. The 6-31G (d, p) basis set was employed for C, H, O and N atoms [4], whereas the LANL2DZ basis set with the related effective core potentials 94, was used for La atoms [5]. Moreover, the time-dependent DFT (TD-DFT) calculations of DFT-optimized ground-state (S_0) geometries were also performed to simulate the UV-vis absorption behavior of the two Ln^{3+} enantiomers, and the related calculated absorption wavelengths (λ/nm), oscillator strengths (f), electronic vertical excitation energies (E/eV), transition types and properties details were established by interfragment charge transfer (IFCT) analysis, and summarized in Table S3.

Synthesis and Characterization of the Pyrazolone Ligand HPMIP (1-phenyl-3-methyl-4-(isobutyryl)-5-pyrazolone)

The pyrazolone ligand **HPMIP** was synthesized according to a modified procedure described in the literature [6]. In more detail, 1-phenyl-3-methyl-5-pyrazolone (**PMP**;

7.5 g, 43.1 mmol) was added to 1,4-dioxane (45 mL) and heated until the solid was completely dissolved. After the solution cooled slightly, $Ca(OH)_2$ (6.0 g, 81.1 mmol) was added to the reaction system, which was further stirred for 10 min. Subsequently, isobutyryl chloride (4.5 mL, 0.04 mol) was added dropwise over 5 min, and the reaction mixture was stirred at 70 °C for 2 h and thereafter cooled to 25 °C. Ice-cold concentrated hydrochloric acid (23 mL, 37%) was added to the mixture, followed by 176 mL of distilled water. The brownish-red precipitate was filtered and purified by recrystallization from CH_3OH and H_2O to give the light-yellow microcrystalline products. The target ligand was prepared *via* the synthetic route presented in Scheme S1. The analysis data for **HPMIP**: Yield = 9.3 g, 88%. Anal. Calcd. for $C_{14}H_{15}N_2O_2$: C, 68.83; H, 6.60; N, 11.47%. Found: C, 68.79; H, 6.58; N, 11.42%. FTIR (KBr, cm^{-1}): 3440 (w), 2972 (w), 2925 (w), 2869 (w), 1619 (vs), 1557 (s), 1495 (s), 1447 (m), 1363 (m), 1315 (m), 1202 (w), 1158 (w), 1061 (w), 1027 (w), 988 (w), 899 (w), 819 (w), 750 (m), 689 (m), 636 (w), 603 (w), 509 (w), 430 (w). ^1H NMR ($CDCl_3$, 400 MHz, 298 K): δ (ppm) 7.82 (2H, m, -Ph), 7.44 (2H, t, -Ph), 7.27 (1H, t, -Ph), 3.15 (1H, m, -CH of $-H^d$), 2.48 (3H, s, $-CH_3$ of $-H^e$), 1.25 (6H, d, $-CH_3$ of $-H^f$).

Synthesis and Characterization of the Series of Dihydrate Ln^{3+} Complex Precursors $[Ln(PMIP)_3(H_2O)_2]$ (Ln = La, Tb, or Gd)

The dihydrate Ln^{3+} complex precursors were synthesized according to the previously reported procedure [6]. To a stirred CH_3OH solution (25 mL) of the pyrazolone ligand **HPMIP** (0.439 g, 1.8 mmol), an equimolar amount of solid NaOH (0.072 g, 1.8 mmol) was added, and the resultant mixture was refluxed for 3 h. Another CH_3OH

solution (15 mL) of Ln^{3+} chloride hexahydrate salt $LnCl_3{\cdot}6H_2O$ (0.6 mmol; Ln = La, 0.212 g; Tb, 0.224 g; Gd, 0.223 g) was added. Each mixture was stirred at 75 °C for 3 h, cooled to room temperature, filtered into a 50 mL beaker, and allowed to naturally volatilize for 3-4 days. This resulted in white block single crystal products of a series of Ln^{3+} complex precursors.

The analysis data for **[La(PMIP)$_3$(H$_2$O)$_2$]**: Yield = 0.467 g, 86%. Anal. Calcd. for $C_{42}H_{49}N_6O_8La$: C, 55.75; H, 5.46; N, 9.29%. Found: C, 55.64; H, 5.52; N, 9.22%. FTIR (KBr, cm^{-1}): 3646 (b), 2984 (w), 2360 (w), 1615 (m), 1499 (m), 1435 (m), 1397 (w), 1363 (w), 1316 (w), 1277 (s), 1088 (w), 1065 (w), 981 (w), 906 (w), 838 (w), 750 (vs), 691 (w), 610 (w), 511 (w). ^{1}H NMR ($CDCl_3$, 400 MHz, 298 K): *δ* (ppm) 8.01 (d, 6H, -Ph), 7.22 (d, 6H, -Ph), 7.06 (s, 3H, -Ph), 3.13 (m, 3H, -CH), 2.30 (d, 9H, -CH_3), 1.03 (d, 18H, -CH_3). MALDI-TOF MS: *m*/*z* 904.452 (100%), $[M-H]^+$.

The analysis data for **[Tb(PMIP)$_3$(H$_2$O)$_2$]**: Yield = 0.460 g, 83%. Anal. Calcd. for $C_{42}H_{49}N_6O_8Tb$: C, 54.45; H, 5.34; N, 9.09%. Found: C, 55.69; H, 5.54; N, 9.22%. FTIR (KBr, cm^{-1}): 3646 (b), 2985 (w), 2360 (w), 1616 (m), 1499 (m), 1434 (m), 1396 (w), 1363 (w), 1316 (w), 1277 (s), 1086 (w), 1064 (w), 982 (w), 905 (w), 840 (w), 751 (vs), 690 (w), 610 (w), 511 (w). MALDI-TOF MS: *m*/*z* 924.367 (100%), $[M-H]^+$.

The analysis data for **[Gd(PMIP)$_3$(H$_2$O)$_2$]**: Yield = 0.443 g, 80%. Anal. Calcd. for $C_{42}H_{49}N_6O_8Gd$: C, 54.65; H, 5.35; N, 9.10%. Found: C, 54.59; H, 5.41; N, 9.03%. FTIR (KBr, cm^{-1}): 3645 (b), 2983 (w), 2359 (w), 1614 (m), 1498 (m), 1435 (m), 1394 (w), 1362 (w), 1316 (w), 1275 (s), 1087 (w), 1063 (w), 982 (w), 906 (w), 839 (w), 750 (vs), 689 (w), 611 (w), 510 (w). MALDI-TOF MS: *m*/*z* 923.356 (100%), $[M-H]^+$.

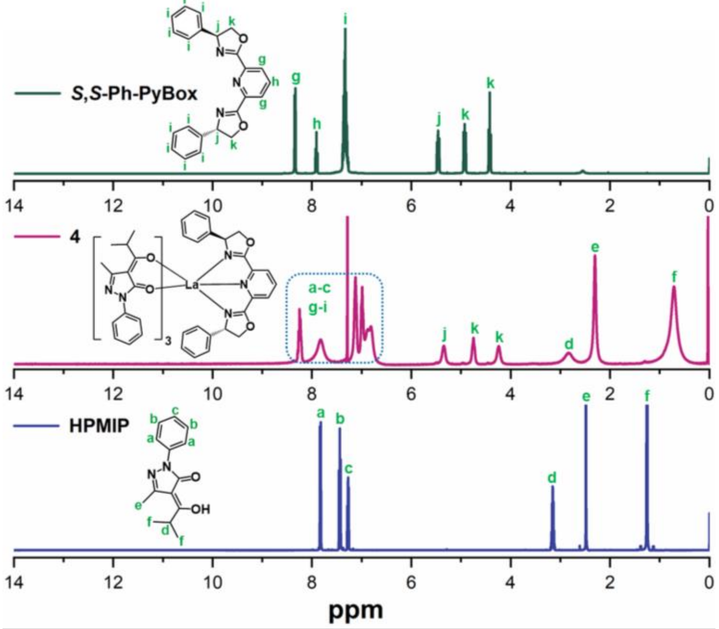

Figure S1. ^{1}H NMR spectra of the chiral La^{3+} complex **4** and associated free *S,S*-**Ph-PyBox** and **HPMIP** ligands in $CDCl_3$ at room temperature.

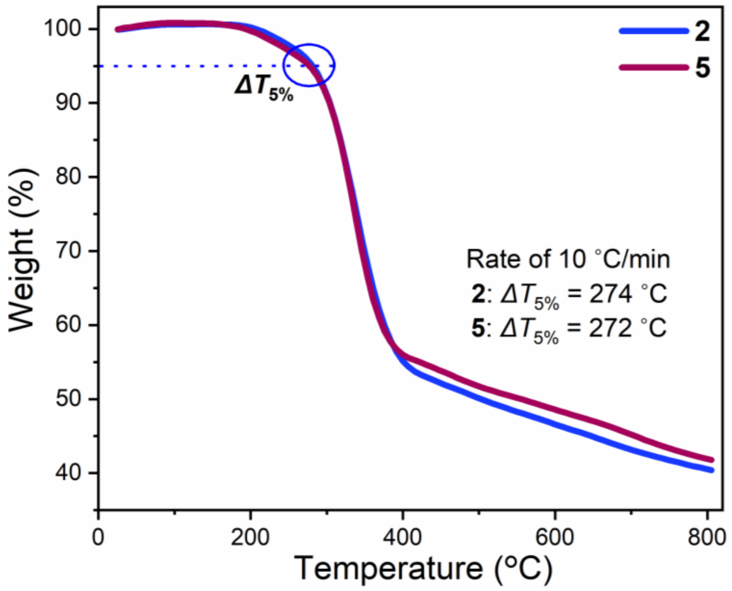

Figure S2. Thermogravimetric (TG) profiles of the chiral Tb^{3+} complexes **2** and **5**.

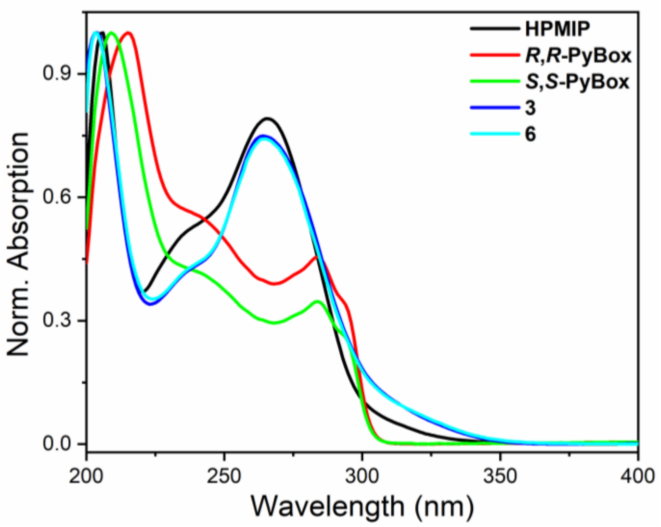

Figure S3. Normalized UV-vis absorption spectra of the pyrazolone **HPMIP** ligand, enantiopure 2,6-bis(4-phenyl-2-oxazolin-2-yl) pyridine ligands (***R*,*R*-Ph-PyBox** and ***S*,*S*-Ph-PyBox**) and chiral Gd^{3+} complexes **3** and **6** recorded in CH_3CN (1×10^{-5} M) at room temperature.

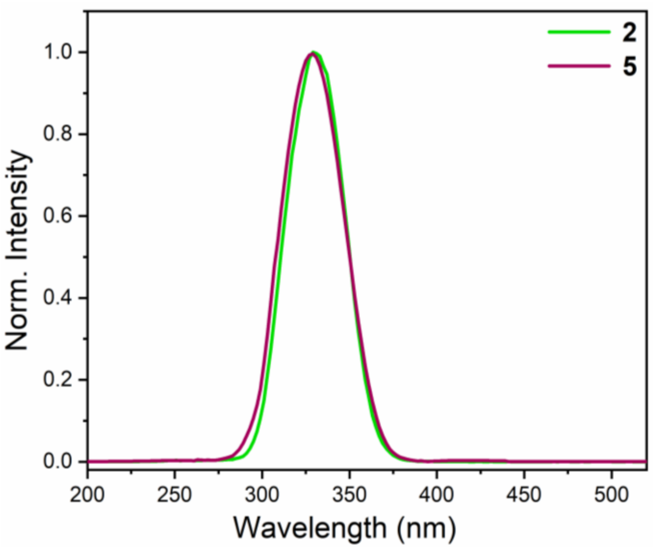

Figure S4. Normalized excitation spectra for the chiral Tb^{3+} complexes **2** and **5** recorded by monitoring emission bands of Tb^{3+} ions at 545 nm in CH_3CN (1×10^{-5} M) at room temperature.

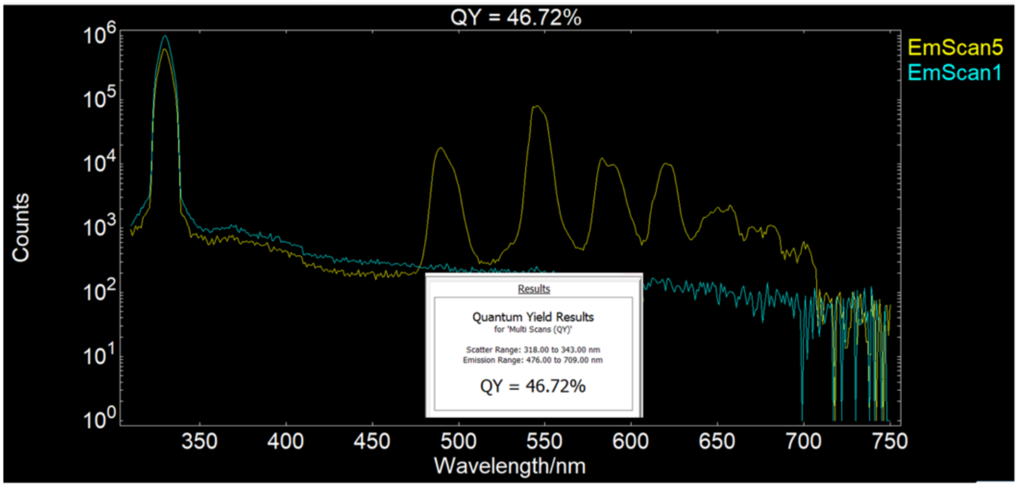

Figure S5. Luminescent absolute quantum yield of the complex **2** based on the overall $^5D_4 \rightarrow {}^7F_J$ (J = 6-0) transition.

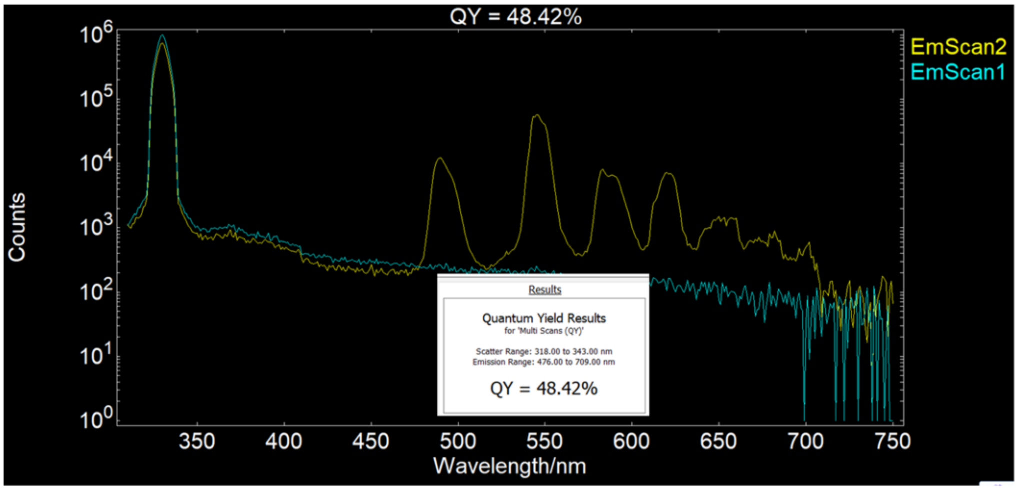

Figure S6. Luminescent absolute quantum yield of the complex **5** based on the overall $^5D_4 \rightarrow {}^7F_J$ (J = 6-0) transition.

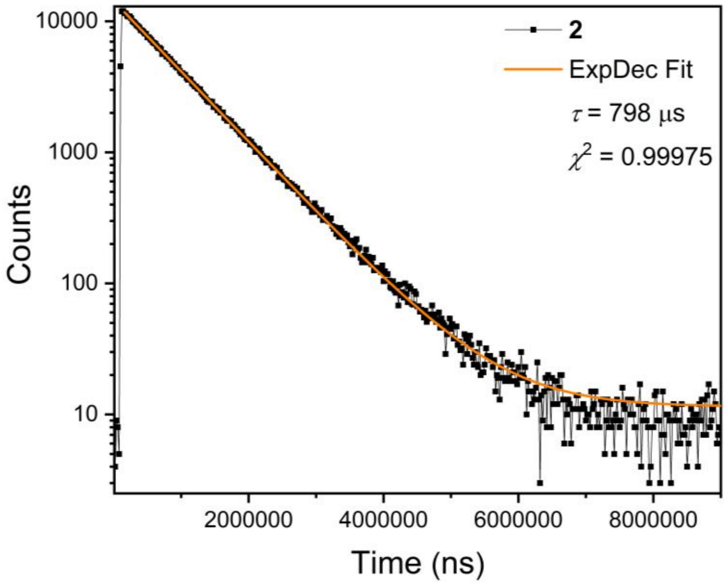

Figure S7. Room temperature decay curves of the complex **2** in CH_3CN (1×10^{-5} M) monitored at 545 nm.

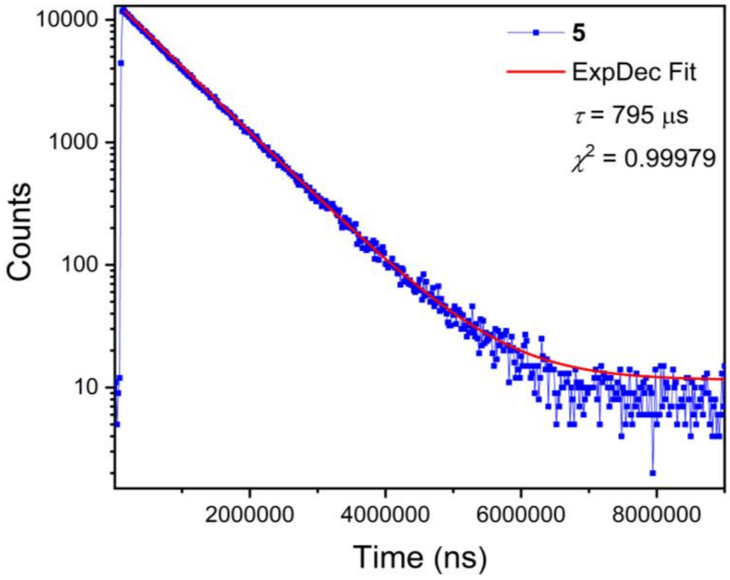

Figure S8. Room temperature decay curves of the complex **5** in CH_3CN (1×10^{-5} M) monitored at 545 nm.

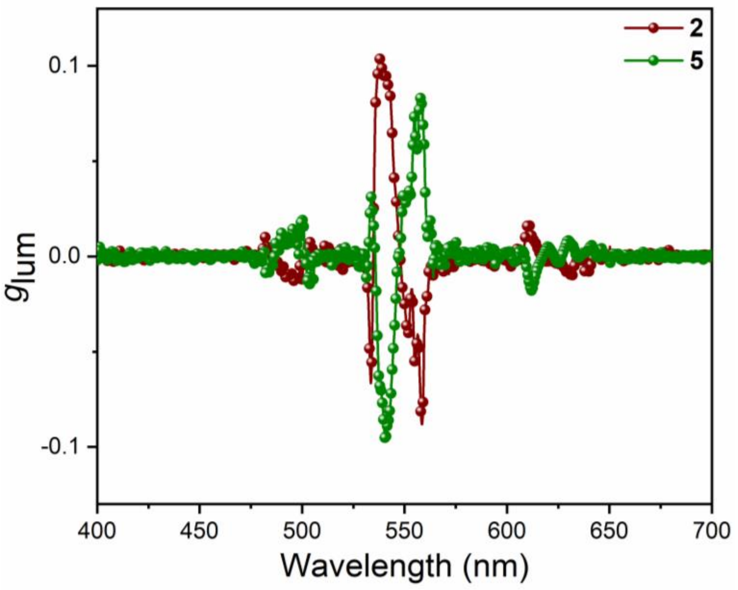

Figure S9. The g_{lum} profiles of the chiral Tb^{3+} complexes **2** and **5** recorded in CH_3CN (1×10^{-5} M) at room temperature.

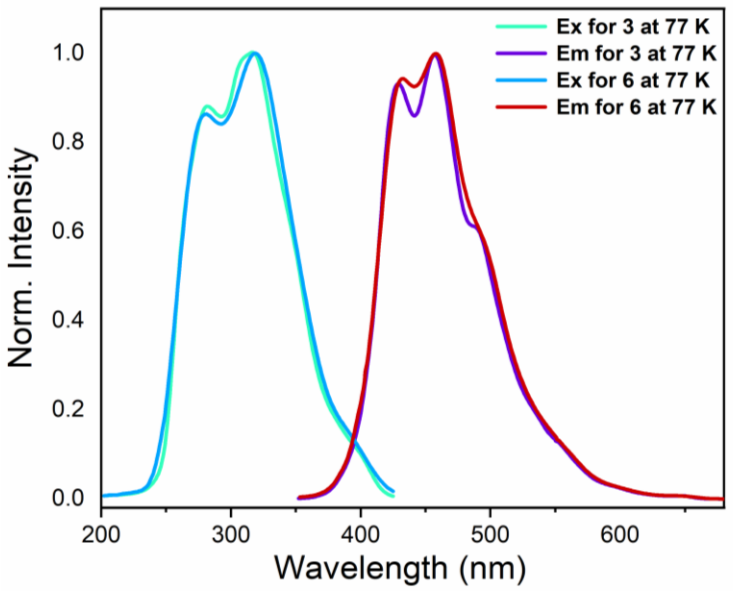

Figure S10. Normalized excitation and emission spectra for the chiral Gd^{3+} complexes **3** and **6** recorded in CH_3CN (1×10^{-5} M) at 77 K.

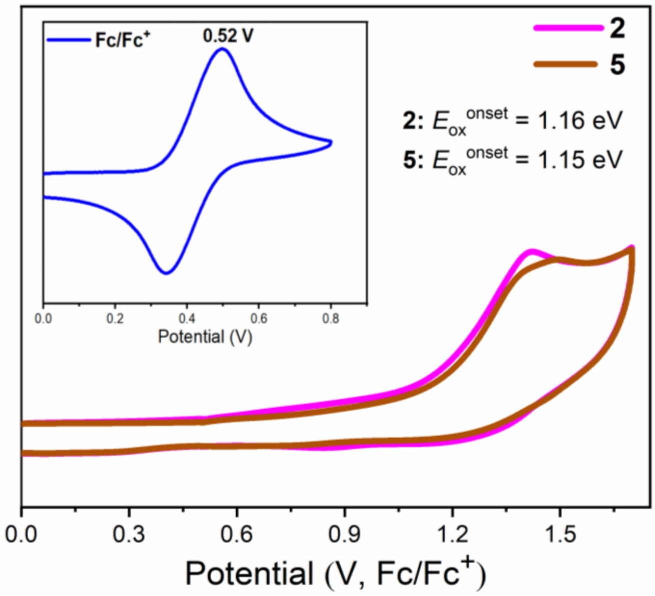

Figure S11. The CV curves of the chiral complexes **2** and **5** recorded as a function of Fc/Fc^+ in degassed CH_3CN solution at room temperature in a N_2 atmosphere (scan rate = 100 mV/s).

Table S1. A comparison of the circularly polarized green luminescence metrics recorded in this work with previously reported chiral heteroleptic binary Tb^{3+} emitters.

Compound	ε_λ (M^{-1} cm^{-1})	Φ_{PL}	$\lvert g_{lum} \rvert$ at $^5D_4 \rightarrow {^7F_5}$	β_i	B_{CPL} (M^{-1} cm^{-1})	Ref.
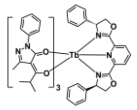	38400-39500 (263 nm)	47-48%	0.096-0.103	67%	610-623	This work
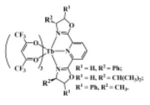	/	Undetectable	0.044-0.082	/	/	[7]
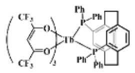	29000 (300 nm)	2%	0.008	67%	1.5	[8]
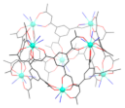	/	0.13%	0.25	/	/	[9]
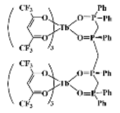	/	2%	0.04	/	/	[10]
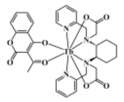	12000 (313 nm)	55%	0.05	55%	11.7	[11]

Table S2. Frontier orbitals and the corresponding electron cloud density distributions for the two chiral La^{3+} complexes on the basis of optimized S_0 geometries.

Compound	Orbital	Contribution of metal d_π orbitals and π orbitals of ligand to MOs (%)				
		**La**	**$(PMIP)^-$-1**	**$(PMIP)^-$-2**	**$(PMIP)^-$-3**	**Chiral-Ph-PyBox**
**1**	LUMO+2	7.74	19.25	0.50	69.78	2.73
	LUMO+1	0.29	0.04	0.11	0.20	99.35
	LUMO	1.15	0.06	0.20	0.51	98.09
	HOMO	0.55	44.03	53.41	1.64	0.37
	HOMO-1	0.73	4.07	11.27	83.32	0.62
	HOMO-2	1.55	50.75	33.85	13.34	0.51
**4**	LUMO+2	7.82	21.77	0.76	67.30	2.35
	LUMO+1	0.31	0.04	0.16	0.21	99.28
	LUMO	1.15	0.04	0.23	0.53	98.05
	HOMO	0.55	45.02	53.23	0.85	0.35
	HOMO-1	0.70	5.34	10.27	83.10	0.59
	HOMO-2	1.62	48.53	34.88	14.46	0.51

Table S3. Electronic vertical excitation energy and the corresponding transition and properties for the two chiral La^{3+} complexes from IFCT analysis of TD-DFT calculations.

Compound	State	λ (nm)	E (eV)	Oscillator (f)	Transition (% contrib.)	Assignment (%)	
**1**	$S_0 \rightarrow S_1$	415	2.2716	0.0006	H→L (97.47);	^{1}LLCT: 97.81	^{1}LMCT: 1.14
						^{1}MLCT: 0.55	^{1}ILCT: 0.51
	$S_0 \rightarrow S_2$	403	2.3398	0.0062	H-1→L (85.82);	^{1}LLCT: 97.10	^{1}ILCT: 1.14
					H-2→L+1 (6.40);	^{1}LMCT: 1.13	^{1}MLCT: 0.63
	$S_0 \rightarrow S_3$	389	2.3796	0.0010	H-2→L (60.21);	^{1}LLCT: 97.28	^{1}MLCT: 1.29
					H→L+1 (31.51);	^{1}LMCT: 0.86	^{1}ILCT:0.57
	$S_0 \rightarrow T_1$	439	2.2677	0.0000	H→L (96.08);	^{3}LLCT: 97.81	^{3}LMCT: 1.14
						^{3}MLCT: 0.55	^{3}ILCT: 0.51
**4**	$S_0 \rightarrow S_1$	416	2.3181	0.0007	H→L (96.38);	^{1}LLCT: 97.82	^{1}LMCT: 1.14
						^{1}MLCT: 0.54	^{1}ILCT: 0.50
	$S_0 \rightarrow S_2$	402	2.3670	0.0063	H-1→L (87.64);	^{1}LLCT: 97.10	^{1}ILCT: 1.16
					H-2→L+1 (5.07);	^{1}LMCT: 1.13	^{1}MLCT: 0.62
	$S_0 \rightarrow S_3$	390	2.4189	0.0010	H-2→L (64.46);	^{1}LLCT: 97.19	^{1}MLCT: 1.35
					H→L+1 (28.73);	^{1}LMCT: 0.89	^{1}ILCT: 0.57
	$S_0 \rightarrow T_1$	438	2.3148	0.0000	H→L (94.86);	^{3}LLCT: 97.75	^{3}LMCT: 1.14
						^{3}ILCT: 0.56	^{3}MLCT: 0.55

Table S4. Summary of voltammetric data of studied chiral complexes **2** and **5**.

Compound	E_{ox}^{onset} (V)	E_{Fc/Fc^+} (V)	E_{HOMO} (eV)[b]	E_g^{opt} (eV)[c]	E_{LUMO} (eV)[d]
**2**	1.16[a]	0.52	-5.44	3.57	-1.87
**5**	1.15[a]	0.52	-5.43	3.57	-1.86

[a]Irreversible or quasi-reversible. The values were derived from the anodic peak potential. [b]HOMO levels are calculated from the equation ($E_{HOMO} = -(E_{ox}^{onset} - E_{Fc}/_{Fc^+} + 4.8)$ eV; where E_{ox}^{onset} is measured in MeCN and reported *versus* the Ag/AgCl). [c]Optical energy gap E_g^{opt} was estimated from the absorption onset using the equation ($E_g^{opt} = 1240/\lambda_{edge}^{onset}$). [d]The LUMO energy levels of each complex were calculated according to the following equation ($E_{LUMO} = E_{HOMO} + E_g^{opt}$).

Supplementary References

(1) Swinehart, D. F. The Beer-Lambert Law. *J. Chem. Educ.* **1962**, *39* (7), 333.

(2) Arrico, L.; Di Bari, L.; Zinna, F. Quantifying the Overall Efficiency of Circularly Polarized Emitters. *Chem. - Eur. J.* **2021**, *27* (9), 2920-2934.

(3) Gong, Y.-J.; Zhang, X.-B.; Mao, G.-J.; Su, L.; Meng, H.-M.; Tan, W.; Feng, S.; Zhang, G. A Unique Approach Toward Near-Infrared Fluorescent Probes for Bioimaging with Remarkably Enhanced Contrast. *Chem. Sci.* **2016**, *7* (3), 2275-2285.

(4) Krishnan, R.; Binkley, J. S.; Seeger, R.; Pople, J. A. Self-Consistent Molecular Orbital Methods. XX. A Basis Set for Correlated Wave Functions. *J. Chem. Phys.* **1980**, *72* (1), 650-654.

(5) Hay, P.; Wadt, W. An Initio Effective Core Potentials for Molecular Calculations. Potentials for the Transition Metal Sc to Hg. *J. Chem. Phys.* **1985**, *82* (1), 270-283.

(6) Liu, J.; Shi, Q.; He, Y.; Fu, G.; Li, W.; Miao, T.; Lü, X. Single-Molecule White-Light of *Tris*-Pyrazolonate-Dy^{3+} Complexes. *Inorg. Chem. Commun.* **2019**, *109*, 107573.

(7) Yuasa, J.; Ohno, T.; Miyata, K.; Tsumatori, H.; Hasegawa, Y.; Kawai, T. Noncovalent Ligand-to-Ligand Interactions Alter Sense of Optical Chirality in Luminescent Tris(β-diketonate) Lanthanide(III) Complexes Containing a Chiral Bis(oxazolinyl) Pyridine Ligand. *J. Am. Chem. Soc.* **2011**, *133* (25), 9892-9902.

(8) Taniguchi, A.; Hara, N.; Shizuma, M.; Tajima, N.; Fujiki, M.; Imai, Y. Circularly Polarised Luminescence from Planar-Chiral Phanephos/Tb(III)$(hfa)_3$ Hybrid Luminophores. *Photochem. Photobiol. Sci.* **2019**, *18* (12), 2859-2864.

(9) Tan, Y. B.; Okayasu, Y.; Katao, S.; Nishikawa, Y.; Asanoma, F.; Yamada, M.; Yuasa, J.; Kawai, T. Visible Circularly Polarized Luminescence of Octanuclear Circular Eu(III) Helicate. *J. Am. Chem. Soc.* **2020**, *142* (41), 17653-17661.

(10) Tanase, T.; Nakamae, K.; Okawa, Y.; Hamada, M.; Matsumoto, A.; Nakajima, T.; Nakashima, T.; Kawai, T. Chiral Dinuclear Eu^{III}, Tb^{III}, and Y^{III} Complexes Supported by *P*-Stereogenic Linear Tetraphosphine Tetraoxide. *Chem. - Eur. J.* **2022**, *28* (8), No. e202104060.

(11) Ruggieri, S.; Mizzoni, S.; Nardon, C.; Cavalli, E.; Sissa, C.; Anselmi, M.; Cozzi, P. G.; Gualandi, A.; Sanadar, M.; Melchior, A. Circularly Polarized Luminescence from New Heteroleptic Eu(III) and Tb(III) Complexes. *Inorg. Chem.* **2023**, *62* (23), 8812-8822.
